# Allogeneic stem cells derived from human exfoliated deciduous teeth (SHED) for the management of periapical lesions in permanent teeth: Two case reports of a novel biologic alternative treatment

**DOI:** 10.15171/joddd.2017.021

**Published:** 2017-06-21

**Authors:** Madu Ghana Shyam Prasad, Juvva Ramakrishna, Duvvi Naveen Babu

**Affiliations:** ^1^Department Pedodontics and Preventive Dentistry, St. Joseph Dental College, Eluru, India; ^2^Department of Biochemistry, St. Joseph Dental College, Eluru, India

**Keywords:** Periapical lesions, permanent teeth, pulp necrosis, bioglass, stem cells, tissue scaffolds

## Abstract

Stem cells are the pluripotent cells that have the capacity to differentiate into other specialized cells. Recently, many experiments have been conducted to study the potentiality of stem cells in the tissue regeneration. We report two cases treated utilizing stem cells from human exfoliated deciduous teeth (SHED) in the management of periapical lesions in permanent teeth. Two normal human deciduous teeth from children, 7‒8 years of age, were collected to isolate stem cells. Two patients, one with periapical pathology alone and the other with periapical lesion along with an open apex in young permanent teeth, were selected for the study. After initial debridement of the root canals, homing of SHED was carried out and the access cavity was sealed using glass-ionomer cement. Clinical examination after 7 days, 30 days, 90 days, 180 days and 365 days revealed no symptoms. Closure of open apex and periapical tissue healing were observed radiographically at one-month review and maintained until 365-day review. Positive response to electric pulp testing was recorded for the treated teeth from the 3- to 12-month follow-ups. The treated cases demonstrated complete resolution of periapical radiolucency in a span of 30 days, which was faster than the conventional methods. SHED could be considred effective in treating the periapical lesions and open apex in permanent teeth.

## Introduction

Recent advances in the stem cell research has created much interest in dental tissue regeneration, resulting in the emergence of tissue engineering in dentistry.^1,2^ Mesenchymal stem cells present in the dental pulp are defined as dental pulp stem cells (DPSCs). DPSCs obtained from human exfoliated decisuous teeth are known as SHED; these cells have the capacity to replicate and renew themselves. The lesions in the periapical area are the sequela of pulpal disease and are diagnosed during the routine radiographic examination.^3^ Various materials and techniques like pure calcium hydroxide, MTA, lesion sterilization and tissue repair, and periapical surgery have been advocated in the past. Traditional calcium hydroxide apexification procedure requires multiple visits and the barrier formed by calcium hydroxide apexification is porous and non-continuous, and makes the tooth brittle due to proteolytic and hygroscopic properties. MTA apexification technique provides non-porous and continuos barrier formation in one or two appointments but it has not been shown to reinforce teeth. Lesion sterilization and tissue repair (LSTR) technique is associated with discoloration of the treated tooth.^4^

Recently, many studies are focussing on the use of stem cells in regenerative medicine.^5^ Hence, this experiment was aimed to evaluate the effectiveness of stem cells from human exfoliated deciduous teeth (SHED) in the management of periapical lesions and open apex in permanent teeth. We here present two cases of periapical lesions that were successfully treated utilizing SHED.

## Case report

### Sources

Two normal human deciduous teeth from children aged 7‒8 years were collected. Teeth with dental caries and pulpal, periapical and periodontal diseases were excluded.

### Methodology

**Figure 1:**
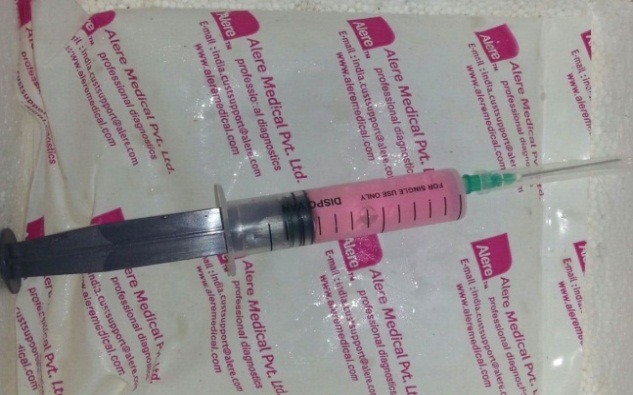
Dental pulp stem cells obtained

**Figure 2:**
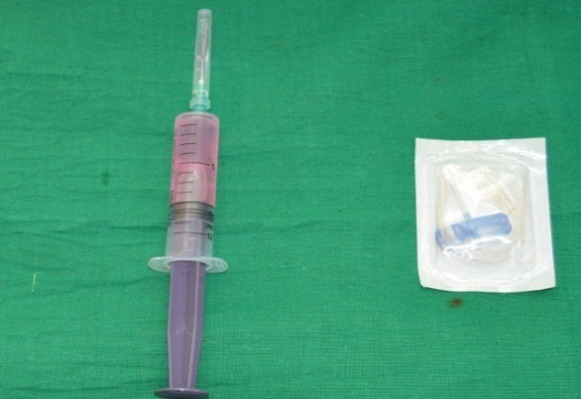
Cells ready for homing.

Enzymatic digestive method of the dental pulp tissue (DPSC-EZ) was used for the isolation of DPSCs.6 This technique involves sterile removel of dental pulp, digestion, characterization and screening by using specific markers. The preparation of culture media, sample collection, storage, handling, expansion, subculturing and characterization of stem cells were performed using the methods described by Vishwanath VR et al.7 The SHED was immediately transported from the laboratory to the department and utilized for the treatment on the same day (Figures 1 & 2).

### Case 1

**Figure 3:**
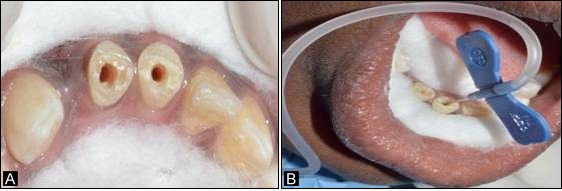
a. Preoperative image after access opening; b. Homing of stem cells in #31 and #41.

**Figure 4:**
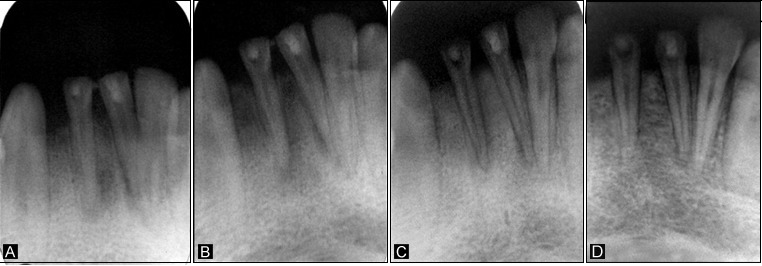
A: Periapical radiolucency in relation to teeth #31 and #41; B: 7-day post-operative radiograph; C: 30-day follow-up radiograph showing complete resolution of radiolucency in relation to teeth #31 and #41; D: 12-month review radiograph.

A twelve-year-old male patient reported to the Department of Pedodontics and Preventive Dentistry, complaining of fractured lower anterior teeth. The patient gave a history of trauma six months back and on clinical examination, an Ellis class IV fracture was seen in teeth #31 and #41. Radiographic examination revealed periapical radiolucency involving both #31 and #41 (Figure 4a). The teeth tested negative to both electric and heat tests. After obtaining parental consent, it was decided to complete the access (Figure 3a) and biomechanical preparation in the first visit, followed by 3Mix (1:1:1 ratio) placement and closure of the access cavity with zinc oxide-eugenol cement. During subsequent appointment after two days, the canals were thoroughly irrigated with normal saline, followed by drying with paper points and placement of tissue scaffold PerioGlas® (bioglass) and homing of SHED from apex to 5 mm of the access cavity with a 25-gauge needle in the root canals of teeth #31 and #41 (Figure 3b). A 5-mm thickness of glass-inomer cement was used to seal the access cavity. The patient was scheduled for recall examination and advised to call if he felt pain. On recall examination after one week, the patient was asymptomatic and reported no pain (Figure 4b). In the 30-day review, the patient was asymptomatic and had no signs of pain or tenderness upon vertical percussion. The radiograph showed significant resolution of radiolucency in relation to teeth #31 and #41 from 30-day recall to the 365-day follow-up (Figure 4C and  4d) and responded positively to electric pulp testing from the 90-day to 365-day recall evaluation. The improvement in the radiolucency may be due to the healing capability of SHED placed in the sterile cavities of teeth #31 and #41. Upon reaching the apical area, SHED might have proliferated and transformed to osteoblast-like cells, leading to bone formation.

### Case 2

**Figure 5:**
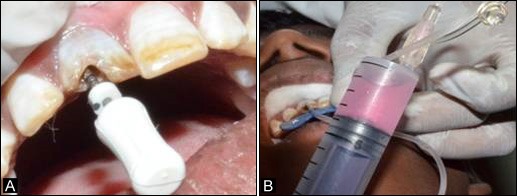
a. Access opening of tooth #11; b. Homing of stem cells into tooth #11.

**Figure 6:**
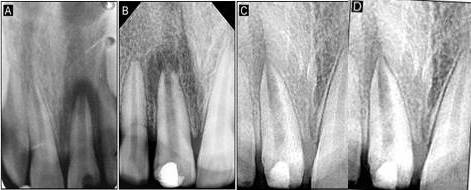
A: Preoperative radiograph displaying periapical radiolucency in relation to tooth #11; B: 7-day postoperative radiograph; C: 30-days follow-up radiograph showing complete radiolucency in relation to tooth #11. D: 365-day follow-up radiograph.

A 12-year-old male patient reported to the Department of Pedodontics and Preventive Dentistry with a chief complaint of fractured upper front tooth. The patient had a history of trauma to tooth #11 two years back. Upon clinical examination, the patient was asymptomatic, and the tooth #11 with Ellis class IV fracture exhibited no response to thermal and electric pulp testing. Radiographically open apex in association with periapical lesion of approximately 2 mm in diameter was seen in #11 (Figure 6a). To promote faster healing, we planned to attempt and achieve regeneration of periapical tissues and pulp by tissue engineering technique with his parental consent. Access opening was prepared and the necrotic pulp tissue was removed (Figure 5a). The root canal was thoroughly irrigated with normal saline solution and dried using absorbent paper points. Then, the scaffold PerioGlas® (bioglass) was introduced into the canal, followed by homing of SHED (Figure 5b) and the cavity was sealed with a glass-ionomer cement of 5 mm thickness. The patient was scheduled for review examination and advised to call if he felt pain. The patient returned after 7 days; he was asymptomatic, reporting no pain to percussion tests (Figure 6b). In the 30-day review, the patient was asymptomatic, and showed no signs of pain. The radiograph showed complete resolution of periapical radiolucency and the closure of apex with thickening of dentinal walls in relation to tooth #11 which continued until the 365-day follow-up period (Figures 6c and 6d). Electric pulp testing was found to be positive for the treated tooth #11 from the 3-month recall to 12-month follow-up.

## Discussion

Infection of the dental pulp is the major cause of periapical pathology leading to the loss of bone and appearance of radiolucency in the radiograph. The main rationale for management is to eradicate the root canal pathogens and substantially reduce the microbial overload. Numerous approaches have been reported in the literature, including non-surgical management, apical surgery and extraction. Initially, periapical lesions are treated with non-surgical conservative methods,^8^ and when such treatments fail, other additional measures must be considered like the repetition of the nonsurgical treatment, surgery or simpler techniques such as marsupialization.

Bhaskar^9^ suggested extension of instruments 1 mm beyond the apical foramen would eventually lead to the development of inflammatory reaction that destroys the cyst lining and thereby, converting the lesion into a granuloma. Upon elimination of the causative factors the granuloma heals spontaneously. Bender explained that penetration into the center of the radiolucency through the apical area helps establish drainage and relieve pressure, which helps in the resolution of the lesion.^10^ Much recently, the Cariology Research Unit of Niigata University advocated ‘Lesion sterilization and tissue repair (LSTR) therapy that involves the use of a combination of antibacterial drugs.' But the above-mentioned treatments require multiple visits and long-term evaluations to assess the clinical success.

The potential role of scaffold as a delivery vehicle for cells has turned out to be progressively essential in a wide assortment of tissues and organs in cellular treatment for local repair. They assume a key role in the activation and attachment of cells in the favorable location. Because of the property of good osteoconductivity they play a major role in avoiding the formation of encapsulating tissue and furthermore helps in the induction of a strong bond between the scaffold and the host bone.^11-13^PerioGlas®, a Bio-active ceramic, was used as a scaffold material in the present case report as it has the advantages of being osteostimulative, osteoconductive and resorbable and also facilitate application.^14^

Literature review provides few promising in vivo investigations of bone repair using dental pulp stem cells (DPSCs). Papaccio’s group showed that autologous DPSCs, when seeded onto collagen sponge scaffolds, could be utilized to repair alveolar defects (d’Aquino et al, 2009).^6^ The autografts which were placed resulted in the repair of the mandibular alveolar bony defects produced after extraction of impacted third molars, producing optimal qualitative and quantitative bone regeneration and bringing about an effective therapeutic strategy.^15^

Yamada et al^16^ analysed DPSCs from canine in an osseointegration study. DPSCs embedded with platelet-rich plasma were placed into bone defects in mandibular canine. Histological perceptions indicated mature, well formed bone, positive for osteocalcin and also signs of neovascularisation, showing the capability of DPSCs for bone regeneration in oral maxillofacial surgery.^16^

In the present study, we used SHED for the treatment of periapical lesions and achieved success in a 30-day span in both the cases. Deciduous teeth are significantly different from permanent teeth in regard to their developmental process, tissue construction and functioning. Therefore the SHEDs have higher rate of proliferation, increased cell population doublings, sphere-like cell cluster formation and osteoinductive capacity factor inducing the host cells to form bone in vivo.^17^ Lee et al^18^ showed the utility of DPSCs to produce dentin/cementum, periodontal ligament and alveolar bone complex.

In our study, both teeth positively responded to cold test and electric pulp test, indicative of re-innervation of the pulp-like tissue within the canal. These findings might be indicative of the presence of pulp or pulp-like tissue within the root canal space after regenerative treatment. Histologic examination of the tissue formed within the root canal space of one case with continued development of root and positive response to electric pulp test at follow-ups revealed presence of a pulp-like tissue.^4,19^

The exact mechanism of healing of periapical lesions is not clearly known, but the possible mechanism of the healing was due to the SHED introduced into the canals reaching the periapical area and differentiating into osteoblast-like cells and thereby promoting adhesion, proliferation, finally leading to osseous regeneration. SHED may also have differentiated into fibroblasts that deposit collagen compressing the capillary network, thereby starving the epithelial cells which are later engulfed by macrophages.

The present case studies were based on the clinical scenario that periapical pathologies are the most frequently encountered diseases in children, causing damage to the healthy pulp that eventually leads to the necrosis and incomplete apical closure. The treatment is often challenging to dentists, as they require long-term treatment periods. Hence, the following study was undertaken to minimize the treatment period.

In the present cases, there were no complications after the homing of stem cells. The patient remained completely asymptomatic from postoperative period until the recent follow-up after 365 days. Although the present cases showed success of SHED in the treatment of periapical lesions and also immature apex, further clinical trials are required to assess the clinical efficiency of SHED.

## Conclusion

Based on the results, the allogeneic SHED as a new biologic alternative might be used for the successful treatment of periapical lesions and open apex in permanent teeth. With this extended knowledge of SHED’s therapeutic properties, further clinical investigations are warranted to assess the histological success. After stem cell banking, the next advance should be the availability of ready-for-use “allogeneic stem cell packs,” which will give dental specialists the capacity to deliver stem cell therapies.

## Acknowledgements

Authors would like to thank Fr. Nelli George, Fr. P. Bala , Dr. Sleeva Raju and staff of the Department of Pedodontics, St. Joseph Dental College, Eluru, Andhra Pradesh, India for their support through out the study.

## Authors' contributions

MGP and DN designed the study and extracted the stem cells from human exfoliated deciduous teeth (SHED). MGP and JRK performed the clinical procedures. MGP, JRK and DN drafted the manuscript. All the authors contributed to critical revision of the manuscript, and have read and approved the final manuscript.

## Competing interests

The authors declare that they have no competing interests with regards to authorship and/or publication of this paper.

## Funding

The authors report no funding for this article.

## Ethical issues

The authors declare that the individuals, whose data were reported in this article, have given written consent to the authors and the Ethics Committee of St. Joseph Dental College, Eluru, Andhra Pradesh, India for the publication of this paper.
